# Microenvironment and Biomarkers in Esophageal Cancers: An Approach for Early Detection and Identification

**DOI:** 10.7759/cureus.74242

**Published:** 2024-11-22

**Authors:** Zakaria Eltahir, Alaa A Eisa, Mohamed H Keayta, Yousif A Aljhani, Omar Alfaroqui, Gareth J Jenkins

**Affiliations:** 1 College of Applied Medical Sciences, Department of Clinical Laboratory Sciences, Taibah University, Medina, SAU; 2 College of Medicine, Translation Clinical Trials Unit, Taibah University, Medina, SAU; 3 Department of Pathology and Laboratory Medicine, Taibah University, Medina, SAU; 4 Department of Histopathology, King Fahad Hospital, Medina, SAU; 5 School of Medicine, Swansea University, Swansea, GBR

**Keywords:** esophagus adenocarcinoma, esophagus cancers, masson trichrome, p63, tumor microenvironment

## Abstract

Background

Esophageal cancer is a prevalent and highly lethal malignancy worldwide, comprising two main subtypes: esophageal squamous cell carcinoma (ESCC) and esophageal adenocarcinoma (EAC). While both subtypes are frequently encountered, ESCC has historically been more common globally. However, in recent decades, EAC has emerged as the predominant type in industrialized nations, often developing from Barrett’s esophagus, a condition driven by chronic gastroesophageal reflux disease (GERD). ESCC typically occurs in the upper esophagus, whereas EAC arises near the lower gastroesophageal junction. The etiology of esophageal cancer is multifactorial, with diverse causes and clinical management strategies. Notably, diagnosing tumors located in the lower esophagus presents significant challenges. EAC is particularly prone to diagnostic errors, as it can be mistaken for ESCC. This study aims to investigate potential histological biomarkers for EAC to facilitate early and accurate identification of histological changes, especially in patients with GERD.

Methods

This cross-sectional study examined archival histological samples from patients with lower esophageal abnormalities. Immunohistochemistry was used to investigate the P63 marker, while Masson’s trichrome stain was employed to evaluate cancer progression and associated microenvironmental changes.

Results

A total of 104 cases were analyzed, including 13 with known P63-positive staining and five P63-negative controls. The remaining cases consisted of both precancerous and cancerous tissues diagnosed as EAC. Among these, 86 cases showed negative P63 staining, confirming their origin from non-squamous cells and supporting their classification as true Barrett’s-related epithelium. In contrast, five of the 13 SCC control cases exhibited P63 positivity, demonstrating a highly significant association (p < 0.00001). Additionally, Masson’s trichrome staining revealed stromal collagen fibers infiltrating the malignant tissue areas.

Conclusions

This study highlights the significance of the P63 marker in distinguishing between ESCC and EAC. It also suggests a potential role for Masson’s trichrome stain in identifying early microenvironmental changes associated with EAC progression.

## Introduction

Esophageal adenocarcinoma (EAC) is a malignancy primarily affecting the distal esophagus and the gastroesophageal junction (GEJ). It is defined as a tumor with its epicenter located within 5 cm of the lower esophagus or the gastric side of the GEJ. This aggressive cancer ranks 11th in global cancer-related mortality [1]. Despite advancements in neoadjuvant chemotherapy and radiotherapy, the prognosis for EAC remains poor [2].

Barrett’s esophagus (BE), a premalignant precursor to EAC, involves a metaplastic transformation of the distal esophageal squamous epithelium into glandular intestinal epithelium. Chronic gastroesophageal reflux disease (GERD), the primary risk factor for BE, causes sustained gastric acid damage to the esophageal lining [3]. The prevalence of BE in the US population is estimated at 8.6%, yet only about 0.5% of affected individuals progress to EAC annually [4-6]. The low progression rate complicates clinical management, as reliable predictors for EAC transition are lacking. Treatment approaches vary widely among clinicians due to the complex evolution of the tumor, posing challenges in locoregional and systemic disease control [7]. Research is urgently needed to improve diagnosis, screening, and surveillance strategies to manage BE and prevent progression to EAC [6-8].

The reasons for the rapid global increase in EAC incidence remain unclear [4]. GERD risk factors include age, gender, alcohol consumption, BMI, lipid profiles, NSAID use, education level, income, marital status, geographic location, and dietary habits, such as consuming hot, sweet, or fatty foods, coffee, or tea [9-11]. Diagnosing BE relies on esophagogastroduodenoscopy, with histological biopsy confirmation. Upper gastrointestinal endoscopy remains crucial for assessing mucosal integrity and detecting early-stage lesions. Despite being the only known risk factor for EAC, BE contributes to a staggering 600% rise in EAC incidence globally over recent decades, affecting both genders [12-14].

Conversely, esophageal squamous cell carcinoma (ESCC) arises from squamous epithelial cells in the esophagus and represents a malignancy capable of widespread metastatic dissemination. SCC can originate in multiple anatomical sites, including the skin, oral cavity, throat, lungs, and genitourinary tract, making it one of the most prevalent metastatic cancers worldwide. Prevention and novel therapeutic strategies are critical for reducing the incidence and mortality associated with ESCC [15].

This study aimed to histologically evaluate esophageal tumors to identify potential biomarkers and morphological changes in their microenvironment. The focus was on early detection and differentiation to improve diagnosis, management, and patient follow-up.

## Materials and methods

This retrospective study analyzed formalin-fixed, paraffin-embedded (FFPE) tissues retrieved from archival samples. A total of 104 gastroesophageal biopsies collected from patients between 2010 and 2018 were included. Endoscopic examinations of these tissues revealed mucosal metaplasia, hyperplasia, or cancer in the lower esophagus, which were subsequently confirmed through histological analysis. Among the biopsies, 79 were diagnosed as EAC, seven exhibited BE or hyperplasia, and 19 displayed early metaplastic changes.

All patients provided informed consent for research participation. The study received approval from the AMS Ethics Committee and the local Health and Research Committee (Project No: 2021/62/105/MLT). Additional ethical approval was granted by the Institutional Review Board of the General Directorate of Health Affairs in Al Madinah Al Munawarah (Certificate No: IRB58-321).

Immunohistochemistry (IHC)

IHC has been developed to complement H&E and special staining techniques, which primarily illustrate tissue morphology. While H&E and special stains often lack specificity in distinguishing tumor subtypes, IHC targets specific protein markers, making it a valuable diagnostic tool for identifying tumors and cytological samples. It has been a standard analytical technique for nearly 50 years [16].

In this study, we performed IHC staining of P63 using a ready-to-use monoclonal antibody supplied by Leica. P63 expression was evaluated in FFPE tissue sections obtained from surgical specimens. Tissue slides, 3-5 μm thick, were incubated at 60 °C for 40 minutes to melt the wax. The staining process was carried out on a fully automated VENTANA BENCHMARK® XT (Ventana, Tucson, AZ, USA) IHC/ISH staining system.

Trichrome stains

Lower esophageal tissue slides (3-5 μm thick) were prepared and processed in a staining container. Deparaffinization was performed using xylene for four minutes, followed by sequential immersion in 100%, 95%, 90%, 80%, and 70% ethanol for four minutes each. The slides were then incubated in warmed Bouin’s solution at 60 °C for 45 minutes and rinsed under running tap water until the yellow tone disappeared.

To separate tissue cores, the slides were immersed in Wiegert’s hematoxylin for eight minutes and washed in running water for five minutes. Cytoplasmic staining was achieved by immersing the slides in anionic dyes and acid fuchsin (C.I. 42590, Merck, Germany) for five minutes, followed by a two-minute rinse with tap water. Slides were subsequently treated with phosphomolybdic acid solution for 10 minutes, then stained with methyl blue (C.I. 42780, Merck) for five minutes to highlight fibroblasts and collagen.

After a two-minute water rinse, the slides were treated with 1% acetic acid water for one minute and dehydrated using ascending ethanol concentrations (70%, 80%, 95%, and 100%), followed by immersion in xylene for 1 minute each. Finally, the slides were mounted with DPX and covered with a coverslip, making them ready for microscopic examination [17].

Statistical analysis

Staining intensities were compared using Fisher’s exact test, with differences considered statistically significant at p < 0.05. Fisher’s exact test evaluates the relationship between the two dimensions of the contingency table (classification into rows vs. classification into columns). The null hypothesis assumes no significant difference between the classifications.

## Results

**Figure 1:**
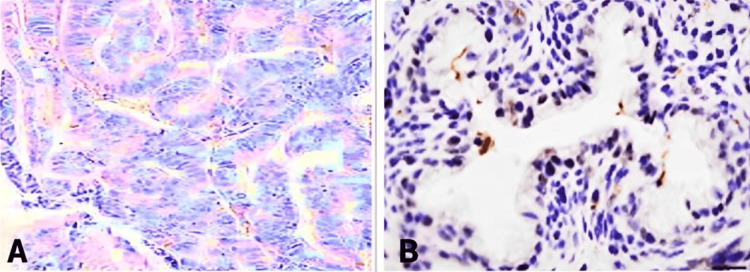
Histology – IHC A representative control staining for negative EAC (A) and positive ESCC (B) P63 IHC. Panel A shows no staining in malignant cells of BE-related tissue, while panel B demonstrates P63 positivity in malignant squamous cells. EAC, esophageal adenocarcinoma; ESCC, esophageal squamous cell carcinoma; IHC, immunohistochemistry

**Figure 2:**
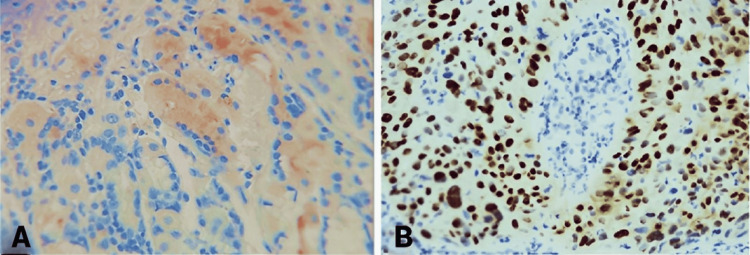
Histology – IHC A representative staining for a P63-negative EAC case (A) showing no reaction, indicating the absence of the P63 antigen in the malignant histology. On the right, a representative histology of positive ESCC (B) with strong antigen-antibody staining. EAC, esophageal adenocarcinoma; ESCC, esophageal squamous cell carcinoma; IHC, immunohistochemistry

**Figure 3:**
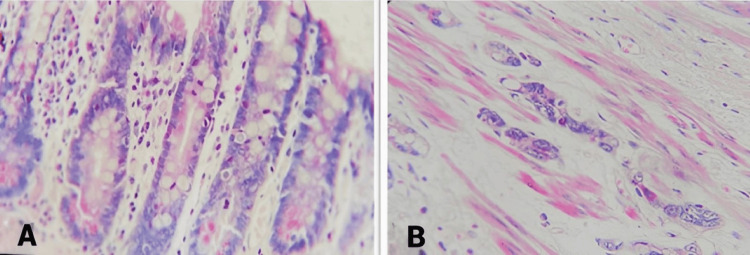
Masson trichrome staining Masson trichrome staining of Barrett’s-related histology showed negative staining (A), with no collagen fibers stained in the non-malignant Barrett’s metaplasia tissue. On the right-hand side (B), the staining reveals a positive fibrosis-like process around a group of malignant cells from BE cancer (EAC), possibly indicating attempts at healing during disease pathogenesis. BE, Barrett’s esophagus; EAC, esophageal adenocarcinoma

This retrospective study analyzed 104 esophageal biopsy samples, including 79 diagnosed with EAC, of which 19 exhibited early metastasis, and seven cases of mucosal metaplasia/hyperplasia (BE). Histological examination confirmed the diagnosis for all 86 cases. Additionally, 18 samples with known P63 antigen expression (13 positive and five negative) were included as quality controls for P63 IHC staining (Table 1). IHC staining intensity was evaluated by two blinded evaluators, unaware of the histological diagnoses. Staining intensity was scored as 1+ for weak expression, 2+ for moderate expression, and 3+ for strong expression, with only 3+ considered positive for P63 immunostaining (Figure 1, Figure 2). Masson trichrome staining, which highlights collagen fibers in the tissue stroma, produced characteristic red and bluish colors (Figure 3). P63 biomarker staining intensities were compared using Fisher’s exact test, with a p-value of less than 0.05 considered statistically significant. This test examines the relationship between the two classifications in the contingency table (rows versus columns), with the null hypothesis stating that no difference exists between the classifications (Table 2).

**Table 1:** Clinical diagnosis data This table presents a detailed overview of the clinical diagnoses related to the biopsies analyzed, both before and after the study. The five cases demonstrating p63 overexpression were all at an advanced stage of cancer. Due to incomplete demographic data, only the information available at the time of the study has been included.

	Study cases			After applying P63	Glandular origin	Squamous origin
	Before the study					
Total gastroesophageal biopsies	86	Malignant	79 (including 19 mets)		74	5
		BE/hyperplasia	7		7	-
Known control P63 - ve	5				5	-
Known control P63 + ve	13				-	13
Total	104				86	18

**Table 2:** Statistical analysis results from Fisher’s exact test The Fisher’s exact test yielded a statistical value of <0.00001, indicating a highly significant result with a p-value less than 0.05. BA, Barrett’s adenocarcinoma; SCC, squamous cell carcinoma

Results			
P63 status	BA	SCC	Marginal row total
P63 - ve	81	5	86
P63 + ve	5	13	18
Marginal column total	86	18	104 (grand total)

Our findings underscore the pivotal role of the P63 biomarker in distinguishing squamous cell tumors from other glandular epithelia, such as those associated with BE. Notably, P63 IHC staining serves as a valuable tool in elucidating the complexities of esophageal cancer. The results demonstrate significant P63 IHC expression across various histological stages, from precancerous lesions to metastatic cancer, suggesting its potential as a marker for tumor identification and improving patient care management. Additionally, Masson trichrome staining revealed distinct morphological changes in the stroma of malignant tumors, facilitating earlier follow-up for patients with GERD who may be at risk for developing EAC, thus enabling timely preventive therapy.

## Discussion

During cancer progression, the morphology of the esophageal epithelium undergoes significant changes, necessitating a comprehensive understanding of these cancers’ biological behaviors to inform diagnosis, prognosis, and therapeutic strategies. Distinguishing between the two types of esophageal cancer is essential, as each demands a unique clinical management approach. Therefore, accurate diagnosis is pivotal to optimizing patient care.

In this study, we assessed the reliability of two distinct markers to address this diagnostic challenge. Specifically, we investigated the role of P63 in differentiating between the two types of esophageal cancer and identifying tumor origin. Additionally, we utilized Masson’s trichrome stain to examine changes in the tumor microenvironment across normal, premalignant, and malignant histologies within EAC. The P63 marker is known to play a crucial role in the development of stratified squamous epithelia, including the esophagus, although its role in glandular Barrett’s-related epithelium remains underexplored [18]. Previous studies have reported a “loss” of P63 expression in BE epithelium [19-21]. Ye et al. specifically highlighted the overexpression of P63 in BE and Barrett’s-associated esophageal cell lines, suggesting its potential role in SCC tumorigenesis through the Akt signaling pathway [22]. Consequently, the absence of P63 expression could provide a reliable means of differentiating EAC.

Our findings support this hypothesis. We investigated 86 cases, including seven precancerous and 79 cancerous biopsies, all histologically confirmed as EAC originating from non-squamous cells. Nuclear immunostaining for P63 was negative in 81 (94.2%) of 86 interpretable BE samples, with five (5.8%) showing strong staining intensity (+3). This difference was statistically significant when compared to non-squamous tissues (p < 0.00001), as determined by Fisher’s exact test, which is appropriate for the limited sample size. Thus, our study suggests that P63 IHC could significantly aid pathologists in accurately diagnosing these cases, reducing the risk of misdiagnosing ESCC or confusing it with Barrett’s cancer [23].

Additionally, the second marker we examined, Masson’s trichrome, revealed new insights into micromorphological changes during Barrett’s cancer progression. Our results showed intense collagen staining occurring exclusively at the cancer stage, with no staining observed in Barrett’s or GERD stages. This observation may highlight potential signaling pathways and help identify patients at risk of developing cancer, facilitating early monitoring of histological changes during the initial phases of carcinogenesis.

An earlier diagnosis of this aggressive cancer is indeed possible, raising questions about the underlying mechanisms driving these microenvironmental changes and collagen fibrogenesis. A review of the literature suggests compelling evidence supporting these observations. Barrett’s disease is primarily associated with GERD, the only known precursor that inflicts significant damage to the lower esophagus, initiating esophagitis [21]. Recent studies emphasize the importance of the cancer microenvironment, particularly in gastrointestinal epithelial cells and cancer stem cells, which respond robustly to their host microenvironment. In the context of Barrett’s disease progression, the host mucosal microenvironment undergoes significant alterations due to the chronic effects of GERD and associated esophagitis [23].

Metaplasia, driven by esophagitis in GERD, marks a critical morphological transition to BE. These cellular changes are unlikely to occur without considerable immune compromise and adaptation. This suggests that the host’s immunological signaling may play a role in promoting carcinogenesis by suppressing anticancer mechanisms, acting as a predisposing factor for cancer initiation [23,24].

Indeed, these fundamental changes are unlikely to occur without significant immune compromise and adaptation. The host’s immunological signaling likely evolves to enable various immune cell types to adapt to the altered microenvironment. In later stages, the host microenvironment may actively contribute to carcinogenesis by suppressing anticancer mechanisms, thus serving as a predisposing factor for cancer initiation and tumorigenesis.

Other researchers have echoed this view, suggesting that understanding these changes could provide opportunities for preventing advanced stages of the disease [24-26]. Gokon et al. noted the presence of different immune cell types at various stages of Barrett’s progression, highlighting CD8+ and T cells as indicators of unfavorable inflammatory responses in this multistep carcinogenic process [27]. Furthermore, Albini and Sporn advocated for the integration of microenvironment studies into chemoprevention strategies, emphasizing their potential in the clinical management of cancer [28].

## Conclusions

Esophageal cancer, particularly ESCC and EAC, presents significant diagnostic and therapeutic challenges due to its high mortality and late-stage diagnoses. Our study underscores the critical role of P63 and Masson’s trichrome as biomarkers for distinguishing between these cancer types and understanding the tumor microenvironment. The absence of P63 expression effectively differentiates EAC from ESCC, while collagen staining patterns offer insights into the progression of Barrett’s cancer. Additionally, the interplay between immune compromise and microenvironmental changes suggests that early intervention could be pivotal in preventing advanced disease stages. Integrating these findings into clinical practice may enhance diagnostic accuracy and inform chemoprevention strategies, ultimately improving patient outcomes in esophageal cancer management. However, the study’s limitations, including the small sample size and lack of comprehensive demographic data, may affect the generalizability of the results.

## Appendices

All data and experiments related to this work are included in the manuscript; if any further details are requested, the authors will be happy to supply them on request.
